# Selective serotonin reuptake inhibitors for fibromyalgia syndrome

**DOI:** 10.1590/1516-3180.20151335T1

**Published:** 2015-08-21

**Authors:** Brian Walitt, Gerard Urrútia, María Betina Nishishinya, Sarah E. Cantrell, Winfried Häuser

## Abstract

**BACKGROUND:**

Fibromyalgia is a clinically well-defined chronic condition with a biopsychosocial aetiology. Fibromyalgia is characterized by chronic widespread musculoskeletal pain, sleep problems, cognitive dysfunction, and fatigue. Patients often report high disability levels and poor quality of life. Since there is no specific treatment that alters the pathogenesis of fibromyalgia, drug therapy focuses on pain reduction and improvement of other aversive symptoms.

**OBJECTIVES:**

To assess the benefits and harms of selective serotonin reuptake inhibitors (SSRIs) in the treatment of fibromyalgia.

**METHODS:**

*Search methods: *We searched the Cochrane Central Register of Controlled Trials (CENTRAL; 2014, Issue 5), MEDLINE (1966 to June 2014), EMBASE (1946 to June 2014), and the reference lists of reviewed articles.

*Selection criteria: *We selected all randomized, double-blind trials of SSRIs used for the treatment of fibromyalgia symptoms in adult participants. We considered the following SSRIs in this review: citalopram, fluoxetine, escitalopram, fluvoxamine, paroxetine, and sertraline.

*Data collection and analysis: *Three authors extracted the data of all included studies and assessed the risks of bias of the studies. We resolved discrepancies by discussion.

**MAIN RESULTS:**

The quality of evidence was very low for each outcome. We downgraded the quality of evidence to very low due to concerns about risk of bias and studies with few participants. We included seven placebo-controlled studies, two with citalopram, three with fluoxetine and two with paroxetine, with a median study duration of eight weeks (4 to 16 weeks) and 383 participants, who were pooled together.

All studies had one or more sources of potential major bias. There was a small (10%) difference in patients who reported a 30% pain reduction between SSRIs (56/172 (32.6%)) and placebo (39/171 (22.8%)) risk difference (RD) 0.10, 95% confidence interval (CI) 0.01 to 0.20; number needed to treat for an additional beneficial outcome (NNTB) 10, 95% CI 5 to 100; and in global improvement (proportion of patients who reported to be much or very much improved: 50/168 (29.8%) of patients with SSRIs and 26/162 (16.0%) of patients with placebo) RD 0.14, 95% CI 0.06 to 0.23; NNTB 7, 95% CI 4 to 17.

SSRIs did not statistically, or clinically, significantly reduce fatigue: standard mean difference (SMD) -0.26, 95% CI -0.55 to 0.03; 7.0% absolute improvement on a 0 to 10 scale, 95% CI 14.6% relative improvement to 0.8% relative deterioration; nor sleep problems: SMD 0.03, 95 % CI -0.26 to 0.31; 0.8 % absolute deterioration on a 0 to 100 scale, 95% CI 8.3% relative deterioration to 6.9% relative improvement.

SSRIs were superior to placebo in the reduction of depression: SMD -0.39, 95% CI -0.65 to -0.14; 7.6% absolute improvement on a 0 to 10 scale, 95% CI 2.7% to 13.8% relative improvement; NNTB 13, 95% CI 7 to 37. The dropout rate due to adverse events was not higher with SSRI use than with placebo use (23/146 (15.8%) of patients with SSRIs and 14/138 (10.1%) of patients with placebo) RD 0.04, 95% CI -0.06 to 0.14. There was no statistically or clinically significant difference in serious adverse events with SSRI use and placebo use (3/84 (3.6%) in patients with SSRIs and 4/84 (4.8%) and patients with placebo) RD -0.01, 95% CI -0.07 to 0.05.

**AUTHORS' CONCLUSIONS:**

There is no unbiased evidence that SSRIs are superior to placebo in treating the key symptoms of fibromyalgia, namely pain, fatigue and sleep problems. SSRIs might be considered for treating depression in people with fibromyalgia. The black box warning for increased suicidal tendency in young adults aged 18 to 24, with major depressive disorder, who have taken SSRIs, should be considered when appropriate.

**The full text of this review is available free of charge from:** http://onlinelibrary.wiley.com/doi/10.1002/14651858.CD011735/abstract
